# The complete chloroplast genome of *Soroseris umbrella* (Asteraceae)

**DOI:** 10.1080/23802359.2019.1711223

**Published:** 2020-01-16

**Authors:** Zhen-Yu Lv, Jian-Wen Zhang, Jun-Tong Chen, Zhi-Min Li, Hang Sun

**Affiliations:** a CAS Key Laboratory for Plant Diversity and Biogeography of East Asia, Kunming Institute of Botany, Chinese Academy of Sciences, Kunming, Yunnan, China;; b School of Life Sciences, Yunnan Normal University, Kunming, Yunnan, China

**Keywords:** Chloroplast genome, phylogeny, *Soroseris umbrella*

## Abstract

*Soroseris umbrella* is an alpine medical plant that is distributed in the alpine screes of China, Bhutan, and India. Here, we identified the complete chloroplast genome of *S. umbrella*. The complete genome size is 152,462 bp, which consists of a large single-copy (LSC) region of 84,125 bp, a small single-copy (SSC) region of 18,561 bp, a pair of inverted repeat (IR) regions of 24,888 bp each. The overall GC content of genome is 37.7%. A total of 114 unique genes were identified, including 80 protein-coding genes, 30 tRNA genes, and 4 rRNA genes. The phylogenetic analysis based on whole chloroplast genome result shows that *S. umbrella* is most closely related to *Taraxacum*.

*Soroseris umbrella* (Franch.) Stebbins is a perennial and medicinal herb characterized by large involucres with 10–15 inner phyllaries, white or yellow florets, and truncate achene apex, which is distributed in the alpine screes of China, Bhutan, and India (Shih 1997; Zhang 2009; Shi and Kilian 2011). This species has even been placed at the monotypic genus *Stebbinsia* as *S. umbrella* Stebbins (Lipschitz 1956) but later some researchers classified it as a member of the *Soroseris* (Bremer 1994; Lack 2007; Maity and Maiti 2007). According to the cytological and molecular work by Zhang et al. (2007, 2011), *Stebbinsia* was confirmed as a section of *Soroseris* (Zhang et al. 2011). Up to now, more than 150 Asteraceae plastomes have been sequenced, a total of four subfamilies (Asteroideae, Cichorioideae, Carduoideae, and Pertyoideae) of the thirteen subfamilies were covered (Lin et al. 2019). However, no complete chloroplast genome of any *Soroseris* species have been recorded. Therefore, we sequenced, assembled, and annotated the whole chloroplast genome of *S. umbrella* and constructed the phylogenetic tree to determine its phylogenetic relationship.

The fresh leaves of *S. umbrella* were collected from Pali, Tibet, China (N27°70′01.41″, E89°18′25.96″). The voucher specimens were stored in KUN (Herbarium, Kunming Institute of Botany, CAS; ZJW3542). The complete chloroplast genome was sequenced using high-throughput sequencing at the Beijing Novogene Bioinformatics Technology Co., Ltd, Beijing, China. The filtered reads were assembled using NOVOPlasty v.3.3 (Dierckxsens et al. 2017). Geseq was used to annotate the plastome genome of *S. umbrella* with the referenced annotations of *Taraxacum mongolicum* at Chlorobox web service (Tillich et al. 2017), and the results were manually corrected using Geneious v.9.0.2 (Kearse et al. 2012). Finally, the sequence and annotations of the *S. umbrella* were submitted to NCBI (GenBank accession number is MN822134).

The complete chloroplast genome length of *S. umbrella* is 152,462 bp, which has a typical quadripartite structure, containing a large single-copy region (LSC; 84,125 bp), a small single-copy region (SSC; 18,561 bp), and two inverted repeat regions (IRs; 24,888 bp each). The GC content is 37.7%. A total of 114 unique genes were annotated, including 80 protein-coding genes, 30 tRNA genes, and 4 rRNA genes. Among these, 7 protein-coding genes (*rpl2*, *rpl23*, *ycf2*, *ndhB*, *rps7*, *ycf15*, and *rps12*), 7 tRNA genes (*trnI-CAU*, *trnL-CAA*, *trnV-GAC*, *trnI-GAU*, *trnA-UGC*, *trnR-ACG*, and *trnN-GUU*), and 4 rRNA genes (*rrn4.5*, *rrn5*, *rrn16*, and *rrn23*) had double copies. Furthermore, 13 genes contain introns, two of which contain two introns (*clpP* and *ycf3*).

The phylogenetic analysis was carried out on the basis of protein-coding sequences of 17 species that belong to the tribe Lactuceae. The sequences were multiply aligned using MAFFT v.7.308 (Katoh et al. 2002) and manually corrected with Geneious v.9.0.2 (Kearse et al. 2012). The phylogenetic tree was reconstructed using maximum-likelihood (ML) estimate with IQtree v.1.6.11 in the IQ-TREE web server (Trifinopoulos et al. 2016). The substitution model was set to auto and bootstrap replicates were 1000. Figtree v.1.4.4 was used to view the ML tree. Our phylogenetic result indicated that *S. umbrella* was a member of Crepidinae (Lactuceae) and was a sister to *Taraxacum* with 100% bootstrap values (Figure 1).

**Figure 1.**
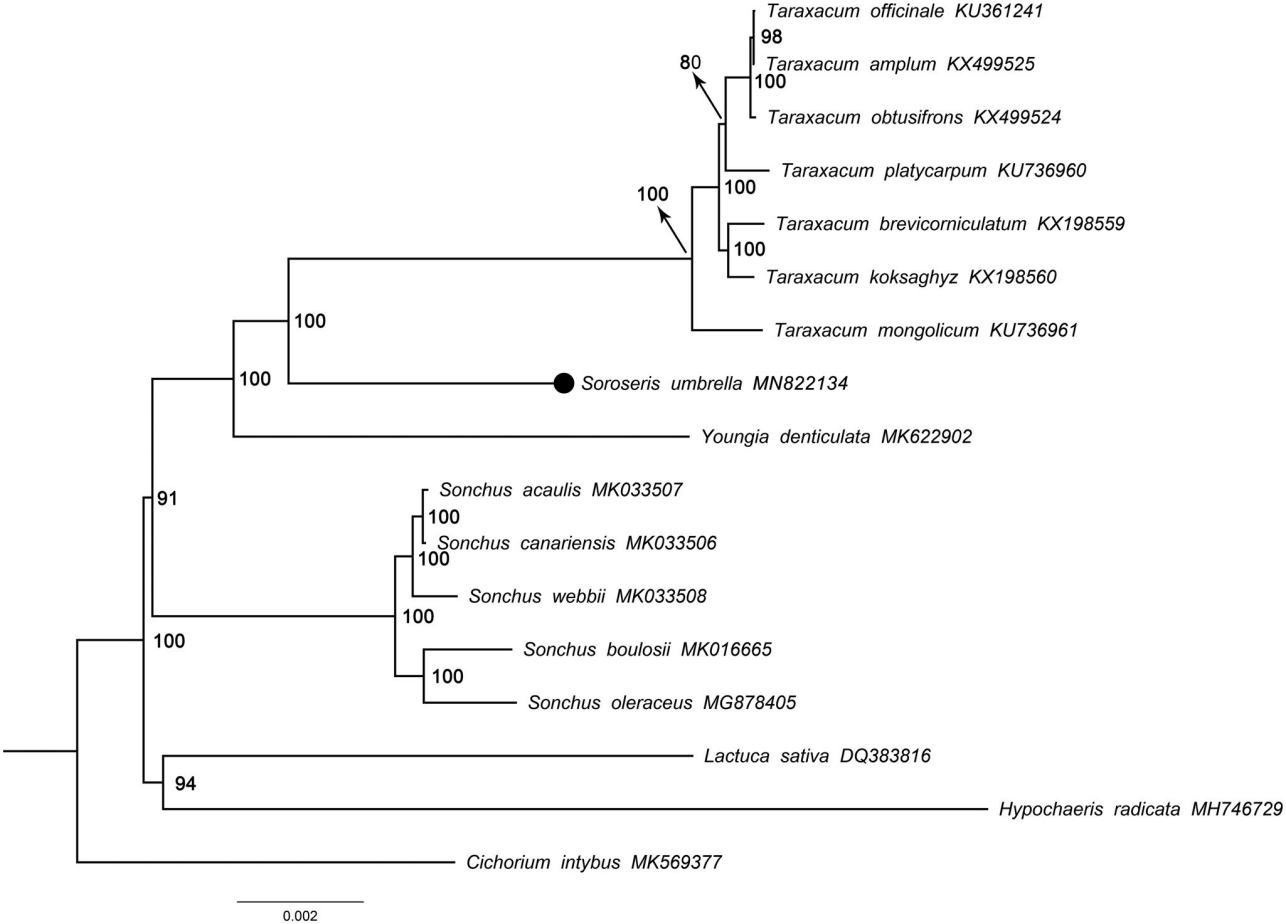
ML phylogeny base on protein-coding sequences of 17 species. The numbers at the nodes are bootstrap values. The black dot indicates *Soroseris umbrella*. GenBank accession numbers are displayed after each species name.
